# Correction: Diagnostic accuracy of digital technologies compared with 12-lead ECG in the diagnosis of atrial fibrillation in adults: A protocol for a systematic review

**DOI:** 10.1371/journal.pone.0362209

**Published:** 2026-11-03

**Authors:** Vethanayagam Antony Sheron, Rajendra Surenthirakumaran, Tiffany E. Gooden, Gregory Y. H. Lip, G. Neil Thomas, David J. Moore, Krishnarajah Nirantharakumar, Balachandran Kumarendran, Kumaran Subaschandran, Shribavan Kanesamoorthy, Powsiga Uruthirakumar, Mahesan Guruparan, on behalf of the NIHR Global Health Research Group on Atrial Fibrillation Management

The fourth author’s initials appear incorrectly in the citation. The correct citation is: Sheron VA, Surenthirakumaran R, Gooden TE, Lip GYH, Thomas GN, J. Moore D, et al. (2024) Diagnostic accuracy of digital technologies compared with 12-lead ECG in the diagnosis of atrial fibrillation in adults: A protocol for a systematic review. PLoS ONE 19(5): e0301729. https://doi.org/10.1371/journal.pone.0301729.
