# Supplementary material for: Diagnostic accuracy of digital technologies compared with 12-lead ECG in the diagnosis of atrial fibrillation in adults: A protocol for a systematic review
Source: PLoS One. 2024 May 8;19(5):e0301729. doi: 10.1371/journal.pone.0301729 (PMC11078345; doi:10.1371/journal.pone.0301729)
Supplement: S2 File — (DOCX) [file pone.0301729.s002.docx]

Additional file 2: Search strategy for MEDLINE

Ovid MEDLINE(R) <1946 to April Week 3 2022>

1 exp Arrhythmias, Cardiac/ (229528)

2 (atrial fibrillation or arrhythmia* or AF or Afib).ti,ab (157564)

3 1 or 2 (285358)

4 exp Telemedicine/ or Electronics, Medical/ or Digital Technology/ or Mobile Applications/ (55510)

5 (mobile app* or mobile phone or smartphone or watch or smartwatch or wristband or wrist band or patch or mobile device or wearable or thumb or single-lead or mHealth or mobile health or digital health or telemed* or telehealth).ti,ab (155955)

6 4 or 5 (185678)

7 3 and 6 (4797)
