# Supplementary material for: Diagnostic accuracy of digital technologies compared with 12-lead ECG in the diagnosis of atrial fibrillation in adults: A protocol for a systematic review
Source: PLoS One. 2024 May 8;19(5):e0301729. doi: 10.1371/journal.pone.0301729 (PMC11078345; doi:10.1371/journal.pone.0301729)
Supplement: Attachment — Submitted filename: Rebuttal letter.docx [file pone.0301729.s003.docx]

University of Jaffna,

Sir. Pon, Ramanathan Rd, Jaffna

Sri Lanka

30^th^ May 2023

Dear Bernadith Millamina,

Manuscript ID number: PONE-D-23-03001

Thank you for reconsidering our manuscript titled “Diagnostic accuracy of digital technologies compared with 12-lead ECG in the diagnosis of atrial fibrillation in adults: A protocol for a systematic review”. We have addressed each comment in a point-by-point format below and have modified the manuscript accordingly. We have uploaded the updated manuscript with tracked changes.

We would be greatly appreciative for your further consideration for publication in PLOS One.

Yours sincerely,

Ms. Vethanayagam Antony Sheron

**Editor comments:**

**Please note that the below comments were provided as ‘additional editor comments’; however, the exact comments, verbatim, were provided from reviewer 4. We have only responded to these comments as the editor’s comments.**

**Comment 1:** Dear Authors, I regret to inform you that the language and sentence structures of this manuscript are at times incomprehensible. The paper needs rewriting and thorough language editing to allow for a proper peer review. First of all, I think there are too many subjective sentences in the introduction part of the article.

**Response 1:** We have thoroughly read the manuscript and have subsequently made amendments to improve the language and sentence structure throughout. Additionally, we have added references to ensure the objectivity of our introduction. We hope you will find these amendments have elevated our manuscript to publication standards.

**Comment 2:** I could not find how many articles were included in this systematic review. And I couldn't find how many articles were excluded and why.

**Response 2:** Our manuscript is a protocol for the systematic review we plan to conduct; we therefore do not have the results of the search to present, nor is this expected to be presented at the protocol stage. As per the Preferred Reporting Items for Systematic Reviews and Meta-Analysis Protocols (PRISMA-P), we have outlined the eligibility criteria in terms of the PICO on page 5-6, lines 108-115 and in Table 1.

**Comment 3:** I could not find any evidence in the references that Digital Technologies are more cost-effective than diagnosis with a 12-lead ECG. Is AF recognition training more expensive in 12-lead ECG?

**Response 3:** Following amendments to the introduction, the following text and references are now included: “These technological advancements are often minimally-intrusive and enable self-testing and ad-hoc long-term monitoring (6); thus, improving early diagnosis and self-monitoring by mitigating barriers of inaccessibility to and non-attendance at healthcare facilities. Advanced devices allow for the transfer of physiological parameters and patient-reported symptoms directly from smartphone applications to healthcare providers, reducing the number of healthcare visits necessary for the public; thus reducing costs on the patient and healthcare system (6,8).”. Both references (listed below), one of which is published in Circulation Research, an American Heart Association journal, are narrative reviews on new and emerging technologies that support our claim of digital technologies being a potential cost-effective strategy for AF diagnosis.

6. Hamad AKS. New technologies for detection and management of atrial fibrillation. J Saudi Heart Assoc. 2021;33(2):169–76

8. Ding EY, Marcus GM, McManus DD. Emerging Technologies for Identifying Atrial Fibrillation. Vol. 127, Circulation Research. Lippincott Williams and Wilkins; 2020. p. 128–42

**Comment 4:** Mobile devices are used a lot in the world, but can these phones be less accessible in low and middle income countries?

**Response 4:** In 2021 half of all inhabitants of LMICs were using mobile internet, a number that has steadily increased over the last 2 decades. We have made amendments to our introduction to emphasise the importance of this exponential increase in mobile usage for understanding the potential benefits of an mHealth tool to diagnose AF in LMICs:

“Mobile connectivity has exponentially increased globally over the last 20 years, including in LMICs where now more than half the population use mobile internet (7,9). As the growth of mobile connectivity continues to rise, the benefits of using digital technologies will become increasingly notable in LMICs where AF burden is increasing the most (10).”

7. Richardson E, Hall A, R.J. Mitchell A. Screening for Atrial Fibrillation and the Role of Digital Health Technologies. Epidemiology and Treatment of Atrial Fibrillation. 2020;1–19

9. Delaporte A, Bahia K. The State of Mobile Internet Connectivity Report 2021. Available from: <https://www.gsma.com/r/somic/> (accessed 2 May 2023).

10. Lippi G, Sanchis-Gomar F, Cervellin G. Global epidemiology of atrial fibrillation: an increasing epidemic and public health challenge. Int J Stroke 2021;16(2):217-21. doi: 10.1177/1747493019897870

**Comment 5:** It has not been reported which of the digital technologies determined as the primary outcome can be used in these countries. I could not conclude which device is the most diagnostic as a result of this article. There is no forest plot in the tables section. In this systematic review, the methodology seems to have been described but not concluded.

**Response 5:** Our manuscript is a protocol for the systematic review we plan to conduct; we therefore do not have the results of the systematic review to present. The methodology is described as per the Preferred Reporting Items for Systematic Reviews and Meta-Analysis Protocols (PRISMA-P).

**Comment 6:** In its current state, I do not recommend accepting this paper.

**Response 6:** We hope you will reconsider with the understanding that most comments are not relevant for a protocol in which we have submitted. Our article was never intended to provide results to a systematic review. We hope you will agree that the remaining comments are minor and that we have appropriately addressed them and made amendments to the manuscript accordingly.

**Reviewer 1 comments:**

**Comment 1:** I think the subject of the article is quite original and interesting. When I read the topic, it really excited me. But I think some definitions are missing.

**Response 1:** Thank you for the positive feedback. We hope you find our amendments and responses below have clarified any confusion around definitions.

**Comment 2:** I couldn't understand how and where to use predictive values.

**Response 2:** The negative and positive predictive values will either be extracted directly from the primary studies included in our systematic review or calculated if the study only provides the true negative and true positive values. We will then report and compare the predictive values for the digital devices included in our review. Please note that the outcome data (including the negative and positive predictive values) for each digital device reviewed will originate from studies where the diagnostic accuracy of the digital device has been compared to a 12 lead ECG (the gold standard).

**Comment 3:** Since I do not know the number of data in the study, I cannot comment on this issue.

**Response 3:** Please note our manuscript is a protocol for the systematic review we plan to conduct; we therefore do not have the results of the systematic review to present.

**Comment 4:** At the same time, the exclusion and inclusion criteria in the study were not clearly explained.

**Response 4:** As per the Preferred Reporting Items for Systematic Reviews and Meta-Analysis Protocols (PRISMA-P), we have outlined the eligibility criteria in terms of the PICO on page 5-6, lines 108-115 and in Table 1. To summarise these criteria: we will include primary cohort and cross-sectional studies that include adults (≥18 years old) and report on the diagnostic accuracy of mobile applications, ECG-based digital technologies or non-ECG-based digital technologies compared to a 12-lead ECG. Only studies that report the following outcomes will be included: sensitivity and specificity, the diagnostic odds ratio (DOR), the negative and positive predictive values (NPV and PPV) or true positive and negative values.

**Comment 5:** Besides, I think that the English used makes it difficult to understand. I think it will be a good interesting article when these are edited.

**Response 5:** We have thoroughly read the manuscript and have subsequently made amendments to improve the language and sentence structure throughout. We hope you will find these amendments have elevated our manuscript to publication standards.

**Reviewer 2 comments:**

**Comment 1:** The inclusion and exclusion criteria should be clear.

**Response 1:** Please refer to response 4 to reviewer 1 above.

**Comment 2:** And better to add an illustration that would demonstrate the protocol and proposed flow of the systematic review.

**Response 2:** We are unsure what kind of flow diagram you are suggesting we should add. Please note that we have mentioned in the ‘analysis’ section that we plan to present the PRISMA diagram of the study selection process in the final systematic review manuscript. However, if there is something more you think is needed at the protocol stage, please let us know and we will consider adding it in.

**Reviewer 3 comments:**

**Comment 1:** Dear Editor, Thank you for giving us the opportunity to review this article titled

“Diagnostic accuracy of digital technologies compared with 12-lead ECG in the diagnosis of

atrial fibrillation in adults: A protocol for a systematic review”. The subject mentioned in the article is as if it was just designed and just remained.

**Response 1:** Thank you. We are unsure what you mean by ‘the subject mentioned in the article is as if it was just designed and just remained.’ If this point could please be clarified, we can address it accordingly.

**Comment 2:** We see that the diagnostic method compared methodologically is not standardized. smart watch, smart belts, holter etc. We could not find any data on which of these devices is more effective in the old literature in which the normal technique was compared.

**Response 2:** Apologies as we are unsure what you mean by ‘normal technique’; however, we will only include studies where the digital device has been compared with a 12-lead ECG (the gold standard) as per our eligibility criteria. From these studies, we will have the sensitivity, specificity, the diagnostic odds ratio (DOR) or the negative and positive predictive values (NPV and PPV) of each digital device to report and compare.

**Comment 3:** The prevalence of smartphone use has been mentioned, but we see that there is no need for additional devices to smartphones for this diagnostic method and data on how widely these devices are used are not mentioned.

**Response 3:** Please refer to response 4 to the editor above.

**Comment 4:** We cannot see an analysis of the cost of the additional devices to be given to each individual and the evaluation made by a trained person in the study.

**Response 4:** Please note our manuscript is a protocol for the systematic review we plan to conduct; we therefore do not have the results of the systematic review to present. We also do not plan to conduct a cost analysis as this is not within the scope of our systematic review. The aim of our systematic review is to assess the diagnostic accuracy of existing digital devices that can be used for diagnosing AF.

**Comment 5:** In addition, this situation is a situation that may be more appropriate in high-income societies, not in low and middle-income countries.

**Response 5:** digital devices are increasingly being used in LMICs as they are often cost-effective and can overcome issues of healthcare access, particularly for rural inhabitants. As mentioned in our introduction (lines 72-82, as below), mobile access is steadily increasing in LMICs, which makes the topic of using digital technologies an increasingly important topic to explore for such settings.

“These technological advancements are often minimally-intrusive and enable self-testing and ad-hoc long-term monitoring (6); thus, improving early diagnosis and self-monitoring by mitigating barriers of inaccessibility to and non-attendance at healthcare facilities. Advanced devices allow for the transfer of physiological parameters and patient-reported symptoms directly from smartphone applications to healthcare providers, reducing the number of healthcare visits necessary for the public; thus, reducing costs on the patient and healthcare system (6,8). Mobile connectivity has exponentially increased globally over the last 20 years, including in LMICs where now more than half the population use mobile internet (7,9). As the growth of mobile connectivity continues to rise, the benefits of using digital technologies will become increasingly notable in LMICs where AF burden is increasing the most (10).”

**Comment 6:** Again, we cannot see the values of the results obtained from the study list included in the meta-analysis in the study text. This obscures the issue of how the study was designed and how the data was obtained and how it was interpreted. This creates the opinion that the evaluation is based on a wish, not on objective parameters.

**Response 6:** Please note our manuscript is a protocol for the systematic review we plan to conduct; we therefore do not have the results of the systematic review to present.
