# Supplementary material for: Diagnostic accuracy of digital technologies compared with 12-lead ECG in the diagnosis of atrial fibrillation in adults: A protocol for a systematic review
Source: PLoS One. 2024 May 8;19(5):e0301729. doi: 10.1371/journal.pone.0301729 (PMC11078345; doi:10.1371/journal.pone.0301729)
Supplement: Attachment — Submitted filename: Kam_Cheong_Wong_review.docx [file pone.0301729.s004.docx]

This proposed systematic review aims to address an important health issue, atrial fibrillation, that is affecting 59.7 million people worldwide. It specifically mentions disparities in access to screening devices in low-middle-income countries (LMICs), which is an important gap in the current literature. If the authors consistently and cogently articulate this point throughout the manuscript, they would have made a strong case for their proposed review. Unfortunately, this point was not consistently carried through, as highlighted in my comments below.

1. The authors have highlighted that low-middle-income countries (LMICs) lack diagnostic equipment and are under-resourced in healthcare systems, generating further barriers. This is a good point. The costs of the screening devices could be barriers. Therefore, the authors may consider adding “device costs” as a parameter in their review. I have noted the authors' reply to Reviewer 3 that the device cost is outside the scope of the review. Paradoxically, the authors added, “As mentioned in our introduction (lines 72-82), mobile access is steadily increasing in LMICs, which makes the topic of using digital technologies an increasingly important topic to explore for such settings”. I perceive “not including device costs in the review” as a wasted opportunity to enhance the consistency and cogency of the authors’ argument that LMICs need affordable screening devices, and the review may generate useful information for LMICs. Of course, this matter is left to the decision of the authors and Editor-in-Chief.
2. The authors wrote, “No one review has reviewed the diagnostic accuracy of all existing devices, making it difficult to compare their accuracy.” This statement provokes arguments because (a) it is almost impossible to review all devices as there is a lack of standardisation and governance in publishing evaluation studies on devices, and (b) it is inappropriate to compare the accuracy of heterogeneous digital devices made of different technologies, such as comparing photoplethysmographic (PPG) devices, oscillometry, mechanocardiographic devices, modified BP meters versus ECG devices that produce rhythm traces. However, it would be more relevant and appropriate if the comparisons were stratified by type of technologies e.g., ECG, PPG, oscillometry etc. The authors can refer to the published systematic review and meta-analysis of the diagnostic accuracy of handheld ECG devices that produced rhythm traces in diagnosing AF (Wong KC, Klimis H, Lowres N, von Huben A, Marschner S, Chow CK. Diagnostic accuracy of handheld electrocardiogram devices in detecting atrial fibrillation in adults in community versus hospital settings: a systematic review and meta-analysis. Heart. 2020 Aug;106(16):1211-1217. doi: 10.1136/heartjnl-2020-316611) and “Giebel GD, Gissel C. Accuracy of mHealth Devices for Atrial Fibrillation Screening: Systematic Review; JMIR Mhealth Uhealth 2019;7(6):e13641”. In addition, the authors cited a series of systematic reviews and meta-analyses in their references 10 to 14. What are the gaps among these reviews? What else are you adding to the literature? The authors should explain how their proposed review addresses the gaps in the current literature.
3. Two reviewers commented about the lack of clarity in inclusion and exclusion criteria. The authors referred them to their existing statements in the manuscript. Perhaps the authors assume inclusion and exclusion criteria are mutually exclusive. However, the authors may think about other exclusion criteria, such as “studies that involved more than one person in interpreting the same ECG findings without reporting the consensus results among the interpreters.”
4. In the second comment, Reviewer 2 asked about the elaboration of the flow of review. The authors replied about including the PRISMA diagram, which is a conventional requirement. I think, in addition to the PRISMA diagram, the authors should elaborate on the number of reviewers in screening titles/ abstracts and how that flows into full-text review (e.g., X number of reviewers check the first N number of full text, resolve discrepancies & reach consensus, then Y number of reviewer proceed with the remaining full-text review).
5. The authors should be aware of potential spectrum effects that have been reported in the literature, i.e., variations of diagnostic accuracy due to differences in the settings in which the devices were used, such as in the community versus the hospital and characteristics of the users. A systematic review and meta-analysis of diagnostic accuracy of handheld ECG devices in diagnosing AF in community and hospital settings reported the variations in AF diagnostic accuracies (doi: 10.1136/heartjnl-2020-316611). Hence, the authors may consider adding the parameter "setting of application" in their review and explore the potential spectrum effect in their reviews.
