# Supplementary material for: Diagnostic accuracy of digital technologies compared with 12-lead ECG in the diagnosis of atrial fibrillation in adults: A protocol for a systematic review
Source: PLoS One. 2024 May 8;19(5):e0301729. doi: 10.1371/journal.pone.0301729 (PMC11078345; doi:10.1371/journal.pone.0301729)
Supplement: Attachment — Submitted filename: Response to Reviewers.docx [file pone.0301729.s005.docx]

15/02/2024

Dear Ernesto Iadanza,

Manuscript ID number: PONE-D-23-03001R1

Thank you for reconsidering our manuscript titled “Diagnostic accuracy of digital technologies compared with 12-lead ECG in the diagnosis of atrial fibrillation in adults: A protocol for a systematic review”. We have addressed each comment in a point-by-point format below and have modified the manuscript accordingly. We have uploaded the updated manuscript with tracked changes.

We would be greatly appreciative for your further consideration for publication in PLOS One.

Yours sincerely,

Dr B. Kumarendran

**Reviewer 1 comments:**

**Comment 1:** Thank you for your revision. I think it is still not clear how and what kind of convenience the use of electronic devices can provide. However, the fact that the subject is new and interesting will guide different studies.

**Response 1:** As described in the introduction section of the revised protocol, electronic devices are low cost compared with the existing medical diagnostic devices for atrial fibrillation (AF) (i.e. 12 lead ECG, ambulatory blood pressure monitors). Furthermore, electronic devices are portable and easily assessable and can be purchased even in low resourced settings for use in primary care or community-based settings which would be beneficial for low-and middle-income countries (LMICs). However, a comprehensive systematic review is needed to find out the hierarchy of diagnostic accuracy of existing electronic diagnostic devices for AF to inform future testing and trials in LMICs.

**Reviewer 2 comments:**

**Comment 1:** The authors have partially addressed our comments. A Research Protocol journal may fit better for the current manuscript.

**Response 1:** We feel we have comprehensively addressed each reviewers’ comments in the revised version of the manuscript; however, if there are any specific comments or suggestions for the protocol that we have not addressed, please kindly provide this feedback and we will consider amending the protocol further. PLOS One accepts study protocols as per their submission guidelines.

**Reviewer 3 comments:**

**Comment 1:** The authors have highlighted that low-middle-income countries (LMICs) lack diagnostic equipment and are under-resourced in healthcare systems, generating further barriers. This is a good point. The costs of the screening devices could be barriers. Therefore, the authors may consider adding “device costs” as a parameter in their review. I have noted the authors' reply to Reviewer 3 that the device cost is outside the scope of the review. Paradoxically, the authors added, “As mentioned in our introduction (lines 72-82), mobile access is steadily increasing in LMICs, which makes the topic of using digital technologies an increasingly important topic to explore for such settings”. I perceive “not including device costs in the review” as a wasted opportunity to enhance the consistency and cogency of the authors’ argument that LMICs need affordable screening devices, and the review may generate useful information for LMICs. Of course, this matter is left to the decision of the authors and Editor-in-Chief.

**Response 1:** We agree the costs of each device is an important factor for use in LMICs; however, costs of any given tool would be specific to a country/region and the use of the tool would be relative to specific average annual incomes to gauge affordability and this will vary even for the same type of tool. Given the nuances here, we will discuss the unit cost and costs implications and options for tools deemed to be of high diagnostic accuracy.

**Comment 2:** The authors wrote, “No one review has reviewed the diagnostic accuracy of all existing devices, making it difficult to compare their accuracy.” This statement provokes arguments because (a) it is almost impossible to review all devices as there is a lack of standardisation and governance in publishing evaluation studies on devices, and (b) it is inappropriate to compare the accuracy of heterogeneous digital devices made of different technologies, such as comparing photoplethysmographic (PPG) devices, oscillometry, mechanocardiographic devices, modified BP meters versus ECG devices that produce rhythm traces. However, it would be more relevant and appropriate if the comparisons were stratified by type of technologies e.g., ECG, PPG, oscillometry etc. The authors can refer to the published systematic review and meta-analysis of the diagnostic accuracy of handheld ECG devices that produced rhythm traces in diagnosing AF (Wong KC, Klimis H, Lowres N, von Huben A, Marschner S, Chow CK. Diagnostic accuracy of handheld electrocardiogram devices in detecting atrial fibrillation in adults in community versus hospital settings: a systematic review and meta-analysis. Heart. 2020 Aug;106(16):1211-1217. doi: 10.1136/heartjnl-2020-316611) and “Giebel GD, Gissel C. Accuracy of mHealth Devices for Atrial Fibrillation Screening: Systematic Review; JMIR Mhealth Uhealth 2019;7(6):e13641”.

**Response 2:** Thank you for the comments. To address point a: We agreed with the reviewer in practical difficulties of capturing all the range of different devices, but we tried to overcome this with subject experts and consultants in the digital technology of AF diagnosis (i.e – Lip G Y ,one of the world’s leading experts in risk stratification of atrial fibrillation, with his research directly informing the widely-used CHA₂DS₂-Vasc and HAS-BLED Scores and Author of 2020 ESC Guidelines for the digital diagnosis of AF), moreover, we have reviewed a list of commercially available digital technology devices on AF diagnosis. And we agree few reviews assessed multiple technologies in a single review, and we amended the gaps in those reviews in the introduction to clarify this point as follows:

“Eight existing systematic reviews have assessed the diagnostic accuracy of digital technologies such as PPG, mobile applications, single lead ECGs and smart wearable devices for AF (12–20). However, six of these reviews only assessed one type of technology. For instance, Belani et al (2021), Gill S et al (2022), Nazarian S (2020) et al and Narut et al (2021) only reviewed the diagnostic accuracy of smart gadgets and wearable devices such smart watches (12, 13, 17, 18), and Wong et al (2020) only reviewed diagnostic accuracy of handheld electrocardiogram (20); and O'Sullivan JW (2020) et al reviewed diagnostic accuracy of smartphone camera applications (16). Including all types of technologies in one review, with pooled diagnostic accuracy stratified by type of device would be useful in deciding which device to take forward for testing in new settings, particular in resource-limited settings. Giebel et al reviewed the diagnostic accuracy of all types of technologies; however, the inclusion criteria were restricted to publication years 2014 to 2019, they did not restrict to studies that only used a 12-lead ECG (the gold standard) as the comparison device. Using different comparison devices can lead to discrepancies in diagnostic accuracy. Lopez et al also reviewed all types of technologies, but also limited their search to 2012 to 2019 and did not restrict to studies using the gold standard as a comparator device (including several studies where the comparator was not described). Furthermore, several studies that report on the AF diagnostic accuracy of digital technologies have been published since the search was conducted for these two existing systematic reviews. Thus, an updated comprehensive review on the diagnostic accuracy of all digital technologies compared to the gold standard will aid in the selection of which digital device(s) can and should be tested in LMICs.” (lines 84-111)

To address point b: We do not plan to pool together all the devices in our review as mentioned in the analysis section of the manuscript: “Where a meta-analysis is possible for any group of primary studies with the same index test (technology), the sensitivity and specificity will be calculated using a random effect model and pooled effects will be presented within the forest plot of individual study estimates”.

**Comment 3:** In addition, the authors cited a series of systematic reviews and meta-analyses in their references 10 to 14. What are the gaps among these reviews? What else are you adding to the literature? The authors should explain how their proposed review addresses the gaps in the current literature.

**Response 3:** The proposed review addresses the following gaps: Majority of systematic reviews assessed the diagnostic accuracy of a single technology or two technologies in their review. Indeed, few systematic reviews assessed the diagnostic accuracy of available digital technologies, however there are few imitations such limitation of publication year, and did not restrict to studies using the gold standard as a comparator device (including several studies where the comparator was not described). We have explained this in the introduction as follows:

“Eight existing systematic reviews have assessed the diagnostic accuracy of digital technologies such as PPG, mobile applications, single lead ECGs and smart wearable devices for AF (12–20). However, six of these reviews only assessed one type of technology. For instance, Belani et al (2021), Gill S et al (2022), Nazarian S (2020) et al and Narut et al (2021) only reviewed the diagnostic accuracy of smart gadgets and wearable devices such smart watches (12, 13, 17, 18), and Wong et al (2020) only reviewed diagnostic accuracy of handheld electrocardiogram (20); and O'Sullivan JW (2020) et al reviewed diagnostic accuracy of smartphone camera applications (16). Including all types of technologies in one review, with pooled diagnostic accuracy stratified by type of device would be useful in deciding which device to take forward for testing in new settings, particular in resource-limited settings. Giebel et al reviewed the diagnostic accuracy of all types of technologies; however, the inclusion criteria were restricted to publication years 2014 to 2019, they did not restrict to studies that only used a 12-lead ECG (the gold standard) as the comparison device. Using different comparison devices can lead to discrepancies in diagnostic accuracy. Lopez et al also reviewed all types of technologies, but also limited their search to 2012 to 2019 and did not restrict to studies using the gold standard as a comparator device (including several studies where the comparator was not described). Furthermore, several studies that report on the AF diagnostic accuracy of digital technologies have been published since the search was conducted for these two existing systematic reviews.” (lines 84 - 111)

**Comment 4:** Two reviewers commented about the lack of clarity in inclusion and exclusion criteria. The authors referred them to their existing statements in the manuscript. Perhaps the authors assume inclusion and exclusion criteria are mutually exclusive. However, the authors may think about other exclusion criteria, such as “studies that involved more than one person in interpreting the same ECG findings without reporting the consensus results among the interpreters.”

**Response 4:** The existing exclusion criteria is provided in Table 1 (please see below); however, we have reviewed it again in light of your comment and have made some minor changes to it. We also recognised that the exclusion criteria were not specifically mentioned within the text; therefore, we have amended the text accordingly (please see below). Thank you for your suggestion regarding consensus results. After discussing with co-authors, we agreed that we will include studies that may or may not report consensus results; however, this is something we will consider when assessing the risk of bias using the QUADAS-2 tool.

Table 1: PICOS of the systematic review

| PICOS | Inclusion criteria | Exclusion criteria |
| --- | --- | --- |
| Population | Adults aged 18 years or above |  |
| Intervention | Mobile application, ECG based digital technology or non-ECG-based digital technology |  |
| Comparison | 12 lead ECG |  |
| Outcome | Sensitivity and specificity, the diagnostic odds ratio (DOR), negative and positive predictive values (NPV and PPV), or true positive (TP), true negative (TN), false positive (FP) and false negative (FN) values for detecting atrial fibrillation | Studies that do not reporting any of these measures for atrial fibrillation will be excluded |
| Study design | Cohort and cross-sectional study designs | Interventional studies, review articles, editorials, case reports, modelling, economic studies, or clinical trials |

“We will exclude any studies that do not report the outcomes of interest specifically for AF and the following study designs: interventional studies, review articles, editorials, case reports, modelling, economic studies, or clinical trials.” (lines 149-151)

**Comment 5:** In the second comment, Reviewer 2 asked about the elaboration of the flow of review. The authors replied about including the PRISMA diagram, which is a conventional requirement. I think, in addition to the PRISMA diagram, the authors should elaborate on the number of reviewers in screening titles/ abstracts and how that flows into full-text review (e.g., X number of reviewers check the first N number of full text, resolve discrepancies & reach consensus, then Y number of reviewer proceed with the remaining full-text review).

**Response 5:** We agree the number of reviewers is important for screening, reviewing of articles and data extraction. This information is already included within the methods section, please see below. Please note this information is also available in the abstract.

“Two reviewers (AS and SK) will independently review titles and abstracts and the full-text of any study where eligibility cannot be determined via the title and abstract. Disagreements will be handled through discussion or a third reviewer (UP).” (lines 162-165)

“Data will be extracted by two reviewers (AS and SK) independently and cross checked for accuracy.” (lines 168-169)

“The Quality Assessment of Diagnostic Accuracy Studies tool (QUADAS-2) will be used to assess risk of bias independently by two reviewers (AS and SK).” (lines 183-184)

**Comment 6:** The authors should be aware of potential spectrum effects that have been reported in the literature, i.e., variations of diagnostic accuracy due to differences in the settings in which the devices were used, such as in the community versus the hospital and characteristics of the users. A systematic review and meta-analysis of diagnostic accuracy of handheld ECG devices in diagnosing AF in community and hospital settings reported the variations in AF diagnostic accuracy (doi: 10.1136/heartjnl-2020-316611). Hence, the authors may consider adding the parameter "setting of application" in their review and explore the potential spectrum effect in their reviews.

**Response 6:** We agree that the setting is extremely important. We have now included within our data extraction methods that we will extract data with regards to setting of device application (grouped under study design/methodology). Additionally, we have also amended our analysis section to include the assessment of setting in terms of application (and not just setting in terms of country-income), please see below.

“We will present the outcomes of sensitivity and specificity using forest plots and will group the studies based on index test first, then by diagnostic threshold of the index test, then by country setting (high-income or low/middle-income) and setting of application (community or healthcare facility).” (lines 199 - 200)

“If heterogeneity is suspected, a Chi-squared test will be performed. Heterogeneity based on sample size (≤100 participants and >100 participants), sampling method (consecutive/convenience), country setting (high-income vs low/middle-income country) and setting of application (community and healthcare facility) will be explored if necessary. If heterogeneity exists but cannot be explained, a meta-analysis will not be performed and results from included studies will be qualitatively synthesized.” (lines 204-211)
